# Hyperuricemia is a Risk Factor for One-Year Overall Survival in Elderly Female Patients with Acute Coronary Syndrome

**DOI:** 10.1155/2020/2615147

**Published:** 2020-02-22

**Authors:** Shi Tai, Xuping Li, Zhaowei Zhu, Liang Tang, Hui Yang, Liyao Fu, Xinqun Hu, Zhenfei Fang, Shenghua Zhou

**Affiliations:** Department of Cardiology, The Second Xiangya Hospital of Central South University, Changsha, China

## Abstract

**Background:**

Hyperuricemia is a risk factor for cardiovascular diseases, but the impact of hyperuricemia and sex-related disparities is not fully clear in elderly patients with acute coronary syndrome (ACS).

**Objective:**

To investigate the association between hyperuricemia and 1-year all-cause mortality in elderly patients with ACS.

**Methods:**

This retrospective cohort study included 711 consecutive ACS patients aged ≥75 years, hospitalized in our center between January 2013 and December 2017. Serum uric acid (sUA), in-hospital events, and 1-year follow-up were analyzed. Multivariable logistic regression models were used to explore the risk factors for in-hospital events and 1-year all-cause mortality.

**Results:**

sUA levels were higher in males than in females (381.4 ± 110.1 vs. 349.3 ± 119.1 *μ*mol/l, *P* < 0.001). Prevalence of hypertension (80.5% vs. 72.6%, *P* < 0.001). Prevalence of hypertension (80.5% vs. 72.6%, *P* < 0.001). Prevalence of hypertension (80.5% vs. 72.6%, *P* < 0.001). Prevalence of hypertension (80.5% vs. 72.6%, *P* < 0.001). Prevalence of hypertension (80.5% vs. 72.6%, *P* < 0.001). Prevalence of hypertension (80.5% vs. 72.6%, *P* < 0.001). Prevalence of hypertension (80.5% vs. 72.6%, *P* < 0.001). Prevalence of hypertension (80.5% vs. 72.6%, *P* < 0.001). Prevalence of hypertension (80.5% vs. 72.6%,

**Conclusions:**

Hyperuricemia is an independent risk factor for 1-year all-cause mortality in elderly female patients with ACS.

## 1. Introduction

The global population aged >60 years is increasing continuously now [1–3], the population aged >60 years is expected to reach 2 billion by the year of 2050 [2]. Aging is natural and will usually lead to physiological changes that compromise the physical, mental, and functional abilities [4]. Elderly population need to be cared for specially and individually due to increased mobility and comorbidity. Diseases features and risk stratification of elderly acute coronary syndrome (ACS) patients might therefore differ from younger patients. ACS is a critical clinical manifestation referring to a wide spectrum of clinical presentations including ST-segment elevation myocardial infarction (STEMI), non-ST-segment elevation myocardial infarction (NSTEMI), and unstable angina [5, 6]. As expected, the prevalence of ACS is significantly associated with aging [7], and the outcome of elderly patients with ACS is also worse in this patient group as compared to younger patients with ACS [8]. In fact, almost one out of two patients hospitalized for ACS is >75 years of age [9]. Despite the application of various risk stratification schemes in patients with ACS [10–13], risk stratification for elderly ACS patients remains challenging due to the lack of sufficient data and reports.

Substantial evidence suggests that serum uric acid (sUA), as the end product of purine metabolism, is an independent biomarker capable of predicting morbidity and mortality in patients with a variety of cardiovascular diseases (CVD) [14], as well as in healthy young and middle-aged adults [15]. The 2018 expert consensus for the diagnosis and treatment of the patient with hyperuricemia [16] highlights the importance of monitoring sUA levels in patients with hyperuricemia and high CV risk [14, 17].

Currently available data are less conclusive regarding the role of sUA as an independent risk factor in elderly patients with ACS. Notably, sex-related disparities in this population remain largely unknown [18]. Therefore, this study aimed to investigate the association between hyperuricemia and 1-year all-cause mortality in elderly patients with ACS, as well as the sex-related differences.

## 2. Methods

### 2.1. Study Design and Patients

This was a retrospective cohort study with 711 patients aged ≥75 years and hospitalized in our center for ACS between January 2013 and December 2017. To ensure exhaustive data collection, only patients who were initially admitted to our center were included; those transferred from other centers were not included. The study protocol conformed to the ethical guidelines of the 1975 Declaration of Helsinki as reflected by prior approval from the human research committee of our institution. The need for individual consent was waived by the committee.

### 2.2. Grouping and Definitions

The study population was divided into two groups according to presence or absence of hyperuricemia. Hyperuricemia was defined as sUA levels of >416 *μ*mol/l (>7 mg/dl) in men and >357 *μ*mol/l (>6 mg/dl) in women [18].

ACS diagnosis was made according to the standard definitions of the American College of Cardiology [5, 19]. In the present study, severe heart failure referred to Killip class III-IV or New York Heart Association (NYHA) class III-IV. Ventricular tachycardia (VT) was defined as more than 5 beats monitored by 24-hour Holter electrocardiogram (ECG).

### 2.3. Data Collection

The following information was collected: body weight and height during hospitalization and calculated body mass index (BMI), diabetes, atrial fibrillation (AF), chronic kidney disease, history of chest pain, history of CVD treatment, smoking, laboratory parameters, hospital stay, demographic characteristics, medication, and in-hospital management and events. sUA levels were routinely measured after an overnight fast from peripheral venous blood samples within the first 24–48 h of hospitalization. The following biochemical parameters were also obtained: hemoglobin, total cholesterol, high-density lipoprotein cholesterol (HDL-C), low-density lipoprotein cholesterol (LDL-C), triglycerides, and glycated hemoglobin A1C (HbA1c).

### 2.4. Follow-Up

In-hospital outcomes were ascertained by hospital chart review. After discharge, participant follow-up was carried out by means of outpatient visits, telephone calls up to one year.

### 2.5. Statistical Analysis

SPSS 23.0 (IBM, Armonk, NY, USA) was used for statistical analysis. Continuous variables are presented as means ± standard deviation, or as median and interquartile range according to their distribution, as determined by the Kolmogorov-Smirnov test; comparisons between groups were performed with the Student's *t*-test or nonparametric tests, as appropriate. Categorical variables are reported as percentage and compared with the chi-square test. Multivariable logistic regression models were used to assess the relationship between risk factors and all-cause mortality. To investigate the possible risk factors for mortality in our study, we first investigated the factors of mortality with univariable analyses based on the clinical characteristics. Multivariable logistic regression analysis was applied with the risk factors defined by univariable analyses with *P* < 0.05. Estimates of odds ratios (ORs) with their 95% confidence intervals (CIs) were reported. *P* values <0.05 were considered statistically significant.

## 3. Results

### 3.1. Characteristics of the Patients

A total of 711 patients were included in the analysis (438 men and 273 women). The sUA concentrations ranged from 107.4 to 942.1 *μ*mol/l (mean ± SD, 369.1 ± 114.6 *μ*mol/l). sUA levels were significantly higher in males than in females (381.4 ± 110.1 vs. 349.2 ± 119.1 *μ*mol/l; *P* < 0.001). There were 182 (25.8%) STEMI and 529 (74.2%) NSTEMI patients; prevalence of hyperuricemia was similar between the two groups (34.1% vs. 35.7%).

### 3.2. Characteristics of the Patients according to sUA

There were 251 (35.3%) patients in the hyperuricemia group and 460 (64.7%) patients in the normal sUA group. The characteristics of each group are listed in Table 1. Body mass index (BMI), prevalence of hypertension, previous chest pain, previous percutaneous coronary intervention (PCI), and stroke were similar between the two groups. The incidence of hypertension (80.5% vs. 72.6%, *P*=0.020), AF (16.2% vs. 9.5%, *P*=0.008), and severe heart function (61.0% vs. 44.2%, *P* < 0.001) were significantly higher in patients with hyperuricemia than in patients with normal sUA.

The clinical features at admission are shown in Table 2. Hemoglobin (119.3 ± 19.2 vs. 113.9 ± 21.6 g/l, *P*=0.001) and HDL-cholesterol (1.1 ± 0.3 vs. 1.0 ± 0.3 mmol/L, *P*=0.001) were significantly lower, whereas creatinine levels (138.0 ± 106.8 vs. 96.6 ± 68.1 *μ*mol/l, *P* < 0.001) and NT-proBNP (887.3 [290.0–2656.2] vs. 1704.0 [536.6–5077.2], *P* < 0.001) were significantly higher in the hyperuricemia group than in normal sUA group.

In-hospital management information is shown in Supplemental [Supplementary-material supplementary-material-1]. Medication was similar between the two groups, except that aspirin (88.6% vs. 78.5%, *P* < 0.001) and diuretics (44.1% vs. 55.9%, *P*=0.003) were used more often in the hyperuricemia group than in the non-hyperuricemia group.

### 3.3. In-Hospital Events and 1-Year Outcomes

In-hospital events are shown in Table 3. There were no significant differences in severe heart failure (15.0% vs. 20.8%, *P*=0.053), bleeding (7.5% vs. 11.1%, *P*=0.113), stroke (1.3% vs. 0.4%, *P*=0.244), and death (3.4% vs. 5.8%, *P*=0.334) between the hyperuricemia and non-hyperuricemia groups, whereas the frequency of VT (11.1% vs. 6.8%, *P*=0.040) was significantly higher in the hyperuricemia group than in the non-hyperuricemia group. Hyperuricemia is related to increased risk of in-hospital VT (unadjusted OR = 1.799, 95% CI 1.050–3.081, *P*=0.033). Besides, hyperuricemia is an independent factor associated with the appearance of AF (OR = 2.118, 95% CI 1.126–3.985, *P*=0.020) after adjustment for confounding variables including age, sex, BMI, current smoking, hypertension, diabetes mellitus, PCI or CABG history, CKD ≥ 3, stroke, ACS type, and severe heart failure. A total of 135 patients (19.0%) died during 1-year follow-up, and the all-cause mortality rate was significantly higher in patients with hyperuricemia than in patients with normal sUA (23.1% vs. 16.7%, *P*=0.039). Patients with hyperuricemia had significantly higher unadjusted hazard of death (unadjusted OR = 1.512, 95% CI 1.028–2.225, *P*=0.036) than patients with normal sUA group. Rehospitalization, revascularization, stroke, and bleeding after discharge were similar between the two groups.

### 3.4. Characteristics of the Patients according to Sex

Sex-related disparities are shown in Table 4. The prevalence of hyperuricemia was significantly higher in females than in males (39.9% vs. 32.4%, *P*=0.042). Female patients had higher frequencies of diabetes mellitus (35.2% vs. 26.5%, *P*=0.014) and NSTE-ACS (79.5% vs. 72.1%, *P*=0.028); lower frequencies of STEMI (20.5% vs. 26.9%, *P*=0.028) and smoking (5.4% vs. 56.5%, *P* < 0.001); higher frequency of severe heart failure on admission (56.8% vs. 46.0%, *P*=0.005); higher total cholesterol (4.1 ± 1.0 vs. 3.8 ± 0.9 mmol/l, *P* < 0.001), HDL-C (1.1 ± 0.3 vs. 1.0 ± 0.3 mmol/L, *P*=0.002), LDL-C (2.4 ± 0.9 vs. 2.2 ± 0.8 mmol/L, *P*=0.002), platelets (202.9 ± 64.9 vs. 174.3 ± 61.4 × 10^9^/L, *P* < 0.001), and HbA1c (median, 6.7% vs. 6.1%, *P*=0.005); and lower hemoglobin (110.4 ± 18.3 vs. 121.8 ± 20.2 g/L, *P* < 0.001), alanine aminotransferase (ALT) (median, 17.5 vs. 19.9 U/L, *P*=0.012), creatinine (89.9 ± 54.1 vs. 124.4 ± 98.6 *μ*mol/L, *P* < 0.001), and sUA (349.2 ± 119.1 vs. 381.4 ± 110.1 *μ*mol/L, *P* < 0.001) than male patients. The in-hospital mortality rate was similar (3.5% vs. 4.4%, *P*=0.693), but male patients had higher 1-year all-cause mortality (21.7% vs. 14.7%, *P*=0.020). Although patients with hyperuricemia had more severe heart failure on admission (66.1% vs. 50.6%, *P*=0.012; 57.0% vs. 40.7%, *P*=0.012, respectively) and higher creatine (111.1 ± 69.2 vs. 76.0 ± 35.3 *μ*mol/L, *P* < 0.001; 158.3 ± 124.4 vs. 108.0 ± 78.5 *μ*mol/L, *P* < 0.001, respectively) and sUA (462.0 ± 101.3 vs. 274.4 ± 52.0 *μ*mol/L, *P* < 0.001; 504.1 ± 84.3 vs. 322.6 ± 61.9 *μ*mol/L, *P* < 0.001, respectively) than those without hyperuricemia in female and male population, respectively, female patients with hyperuricemia had a higher frequency of CKD ≥ 3 (9.2% vs. 1.8%, *P*=0.005), lower EF value (51.6 ± 11.7 vs. 55 ± 9.2%, *P*=0.009), and higher 1-year all-cause mortality (22.0% vs. 9.8%, *P*=0.005) than those without hyperuricemia, but mortality was similar in male patients with or without hyperuricemia.

### 3.5. Multivariable Analyses

We examined the independent determinants of 1-year all-cause mortality. As shown in Supplemental [Supplementary-material supplementary-material-1], female patients with hyperuricemia had significantly higher unadjusted odds of death (unadjusted OR = 2.612, 95% CI 1.315–5.189, *P*=0.006) than female patients with normal sUA. Similar results were observed (OR = 2.438, 95% CI 1.215–4.894, *P*=0.012) after adjusting for age (Model 1). The same associations were observed in model 2 (OR = 2.643, 95% CI 1.092–6.400, *P*=0.031) after adjustment for age, BMI, current smoking, hypertension, and diabetes mellitus and in model 3 (OR = 2.539, 95% CI 1.001–6.453, *P*=0.050) after adjustment for age, BMI, current smoking, PCI or CABG history, hypertension, diabetes mellitus, CKD ≥ 3, and stroke. The multivariable analysis showed that hyperuricemia was not an independent risk factor of 1-year all-cause mortality for male patients and for the total cohort (OR = 0.931, 95% CI 0.466–1.858, *P*=0.839; OR = 1.326, 95% CI 0.773–2.273, *P*=0.305, respectively).

## 4. Discussion

Hyperuricemia is a risk factor for CVDs, but the impact of hyperuricemia and sex-related disparities are not fully clear in elderly patients with ACS. Therefore, this study aimed to investigate the association between hyperuricemia and outcome in elderly patients with ACS. The results suggest that patients with hyperuricemia have higher in-hospital events and 1-year all-cause mortality compared with those without hyperuricemia. Importantly, hyperuricemia is an independent risk factor for one-year all-cause mortality after discharge in elderly female patients with ACS, but not in elderly male patients with ACS.

CVDs are the leading causes of death worldwide [20]. The high mortality in elderly patients with ACS [1, 8] justifies the cumulative risk factors, which might result in poor outcome in this patient population. The present study showed a rate of 19.0% for 1-year all-cause mortality in elderly patients with ACS, which is consistent with a previous report [21]. A large body of evidence indicates that hyperuricemia is an emerging CVD risk factor [22, 23], besides traditional risk factors for CVD. The results of the present study showed that hyperuricemia is associated with the female sex, arterial hypertension, AF, lower HDL-cholesterol, elevated serum creatinine, and in-hospital cardiac complications. It is likely that the higher in-hospital event and one-year all-cause mortality rates after discharge are the consequences of the more frequent clustering of these risk factors, and hyperuricemia might thus be viewed as a surrogate risk factor in elderly ACS patients. The multivariable analyses showed that hyperuricemia is an independent determinant of one-year all-cause mortality in female ACS patients, but not in males or in the whole cohort after adjustment for age, BMI, current smoking, PCI or CABG history, hypertension, diabetes mellitus, CKD ≥ 3, and stroke, as supported by previous studies [24–27]. A previous report from a large Chinese cohort showed that the prevalence of hyperuricemia was 42.6% in men and 23.4% in women [18]; we found that the prevalence of hyperuricemia is even higher in elderly female patients with ACS than in their male counterparts. This pattern confirms the presence of sex-specific differences in elderly patients with ACS in terms of hyperuricemia. This discrepancy could be attributed to menopausal women having higher sUA levels than premenopausal women [28]. Women with ACS, especially at age ≥75, have been described as being sicker than their male counterparts [29]. Similarly, our data also indicate that elderly female patients had more risk factors for CVD, including diabetes mellitus, hypertension, and dyslipidemia. Nevertheless, male patients had worse 1-year outcomes compared with those of women. The possible reason might be that male elderly ACS patients have other special risk factors, such as higher frequencies of STEMI, current smoking, and worse renal function, which may influence the 1-year all-cause mortality. It was shown that the prevalence of hyperuricemia in general population was 21.4% (21.2% for men and 21.6% for women) according to the United States National Health and Nutrition Examination Survey (NHANES) study [30]. It was also shown that the prevalence of hyperuricemia increased with aging in male and female (14.2% at age <30, 20.1% at age 31–40, 22.9% at age 41–50, 22.3% at age 51–60, 23.3% at age 61–70, and 24.1% at age >70 in men; 2.8% at age<30, 3.5% at age 31–40, 6.6% at age 41–50, 14.7% at age 51–60, 16.8% at age 61–70, and 23.4% at age >70 in women) [31, 32]. Our study showed that hyperuricemia served as an independent risk factor in elderly female patients with ACS, implicating the importance of monitoring sUA levels in elderly female ACS patients. Future studies are warranted to verify the prognostic importance of hyperuricemia in ACS patients with various ages and sexes. Moreover, it is of importance to observe that more intensive care, including targeting blood uric acid levels on outcome among elderly female ACS patients, is required.

NHANES III trial showed an increased risk of all-cause death and cardiac death in patients with increased sUA levels, and the association remained significant even after adjustment for different factors including demography and comorbidities [33]. On the other hand, our study showed that hyperuricemia was not associated with in-hospital death and 1-year death in elderly ACS patients. The reason for these discrepancies remains elusive. Although hyperuricemia is a risk factor for 1-year all-cause mortality in the present study, we failed to confirm the independent association between hyperuricemia and 1-year all-cause mortality after adjustment for age and other risk factors, suggesting that, in elderly ACS patients, other comorbidities might serve as more important causes of death than hyperuricemia. Notably, few studies have clarified the relationship between hyperuricemia and sex-specific differences in the outcomes of elderly patients with ACS. Our data indicated that female patients with hyperuricemia have higher frequency of CKD ≥ 3, lower EF value, and higher 1-year all-cause mortality than those without hyperuricemia, but this is not observed in males. Importantly, hyperuricemia was associated with an increased risk of 1-year all-cause mortality in elderly women independently of traditional CVD risk factors, but this similar association was not observed in men and in the total population. These findings suggest that hyperuricemia is associated with 1-year all-cause mortality more strongly in women than in men with ACS. A previous study reported that changes in sUA levels in menopausal women is associated with sex hormones [34], thereby sex hormones may partly explain the different risk of hyperuricemia in males and females. Given that the mechanisms behind these sex-specific differences in ACS remain unclear, the representation of women in clinical CVD trials needs to be increased.

The mortality rate was higher in men than in women, which is consistent with the general CVD mortality pattern observed between men and women [3, 20, 29]. Our findings indicate that, despite lower 1-year mortality among elderly female ACS patients as compared to elderly male ACS patients, hyperuricemia is still considered as an independent risk factor of one-year mortality in elderly female patients, and future studies are warranted to see if more intensive care including therapy targeting hyperuricemia could further reduce the one-year mortality for elderly female ACS patients. Besides, lifestyle habits may also play a role in this difference in mortality. Indeed, in the present study, men had a higher frequency of current smoking than women (57% vs. 5%), which is consistent with the pattern observed in the Chinese population [35]. Smoking is a well-known risk factor for all-cause and CVD death [35]. In addition, men had a higher frequency of STEMI, while women had a higher frequency of severe heart failure. Although the difference was small (but significant), women had higher levels of total, LDL-C and HDL-C than men, but the higher levels of HDL-C probably counteracted the higher LDL-C levels, but this will have to be verified more specifically. Recent evidence suggests that diabetes has a more significant impact on CVD mortality in women than in men [36], but in the present study, mortality in women was lower than in men despite the frequency of diabetes being higher in women; this will have to be investigated.

Lately, a considerable number of reports associating elevated sUA with cardiac rhythm disorders (such as AF) have been published [37, 38]. In the present study, the result indicated that hyperuricemia was an independent factor associated with the frequency of AF (OR = 2.118, 95% CI 1.126–3.985, *P*=0.020) after adjustment for confounding variables. Gang Huang et al. [39] investigated hyperuricemia and the risk of AF in the very elderly community and found that participants with hyperuricemia had a higher risk of suffering from AF in very elderly Chinese. Leonardo Tamariz et al. [40] identified 1,085 cases of incident AF. In Cox proportional hazards models adjusted for age, gender, race, center, education, and traditional risk factors at baseline, the hazard ratio of AF associated with a SD increment in sUA was 1.16 (95% confidence interval 1.06 to 1.26). These results indicated that elevated sUA is associated with a greater risk of AF. It is generally accepted that both inflammatory-dependent and -independent mechanisms might play a role in rhythm disorders [41, 42]. Elevated concentrations of sUA via xanthine oxidase system activation are related to the generation of oxidative stress and inflammatory mediators [16]. Oxidative stress promotes calcium overload and decreases sodium channels, resulting in electrical remodeling [43]. Proinflammatory molecules induced by oxidative injury are also increased [44]. At the same time, structural remodeling may be mediated by fibroblasts [43]. Subsequently, electrical and structural remodeling may lead to AF [45]. Regarding the association between increased sUA levels and ventricular arrhythmias, the evidence is sparse. The present study strongly suggests that patients with hyperuricemia had higher odds than those with normal sUA according to the univariable logistic regression analyses, and this association was lost after adjusting for other confounders. A small observational study of individuals with ECG evidence of hypertrophy divided into two groups based on whether VT was detected or not on a 24-h ECG recording, demonstrated that sUA was an independent factor associated with the appearance of VT [46], but one should keep in mind that arrhythmia has a complex pathophysiologic background. The limited available data only explain the possible relationship and conceptual framework, and additional research may be needed to clarify the underlying mechanisms.

### 4.1. Limitations

This study had some limitations. First, this is a single center experience and the number of patients was relatively small when considering the high incidence of ACS. Therefore, we set a strict standard for the selection of potential variables (*P* < 0.05) for the multivariable logistic analysis, and we might have missed some factors. Besides, the observational study design allows determining only associations and not causality; the mechanisms behind the results obtained require further investigation. Finally, STEMI and NSTE-ACS patients are quite heterogeneous regarding symptoms and outcomes, yet these patients were not analyzed separately in this study.

## 5. Conclusion

Hyperuricemia is associated with in-hospital events and 1-year all-cause mortality, and it is an independent risk factor for 1-year all-cause mortality after discharge in elderly female patients with ACS. Monitoring sUA levels would be helpful to clinicians treating these elderly patients suffering from hyperuricemia and ACS.

## Figures and Tables

**Table 1:** Baseline characteristics of patients according to sUA levels.

Parameters	sUA normal (*n* = 460)	Hyperuricemia (*n* = 251)	*P*
*Demographics and medical history*			
Age, years (median, IQR)	78 (76–80)	78 (76–81)	0.308
Female sex, *n* (%)	164 (35.7)	109 (43.4)	0.042
Body mass index (median, IQR)	22.8 (20.8–25.0)	23.4 (20.8–26.3)	0.101
Diabetes mellitus, *n* (%)	138 (30.0)	74 (29.5)	0.885
Hypertension, *n* (%)	334 (72.6)	202 (80.5)	0.020
Atrial fibrillation, *n* (%)	43 (9.5)	40 (16.2)	0.008
CKD ≥ 3	17 (3.7)	22 (8.8)	0.005
Stroke, *n* (%)	70 (15.5)	33 (13.5)	0.481
Previous chest pain, *n* (%)	299 (65.9)	168 (68.3)	0.514
Previous PCI, *n* (%)	82 (18.1)	32 (13.0)	0.082
Previous CABG, *n* (%)	9 (2.0)	4 (1.6)	0.739
Current smoking, *n* (%)	171 (38.5)	82 (34.2)	0.261

*Examination findings*			
SBP, mmHg (median, IQR)	134 (117–152)	132 (118–152)	0.520
DBP, mmHg (median, IQR)	72 (65–81)	71 (64–80)	0.184
Heart rate, beats/min (median, IQR)	75 (67–84)	76 (68–86)	0.158

*Clinical presentation*			
Severe heart failure, *n* (%)	203 (44.2)	153 (61.0)	<0.001
STEMI, *n* (%)	120 (26.1)	62 (24.7)	0.686
NSTE-ACS, *n* (%)	340 (73.9)	189 (75.3)	0.686

sUA: serum uric acid; IQR: interquartile range; CKD: chronic kidney disease; PCI: percutaneous coronary intervention; CABG: coronary artery bypass graft; SBP: systolic blood pressure; DBP: diastolic blood pressure; STEMI: ST-elevated myocardial infarction; NSTE-ACS: non-ST-elevation acute coronary syndrome.

**Table 2:** Clinical data on admission of patients according to sUA levels.

Clinical data	sUA normal (*n* = 460)	Hyperuricemia (*n* = 251)	*P*
WBC, 10^9^/L (mean ± SD)	7.2 ± 3.2	7.5 ± 3.2	0.252
Hemoglobin, g/L (mean ± SD)	119.3 ± 19.2	113.9 ± 21.6	0.001
Platelets, 10^9^/L (mean ± SD)	188.7 ± 64.2	179.0 ± 64.0	0.058
Fasting glucose, mmol/l (median, IQR)	5.9 (4.8–7.6)	5.6 (4.8–6.7)	0.284
HbA1C, % (median, IQR)	6.3 (5.7–6.9)	6.1 (5.6–6.7)	0.313
ALT, U/l (median, IQR)	18.8 (12.8–31.8)	18.8 (12.7–33.3)	0.948
AST, U/l (median, IQR)	22.9 (17.3–53.2)	22.3 (18.1–37.0)	0.955
Albumin, g/l (mean ± SD)	35.1 ± 4.0	35.0 ± 4.6	0.688
Triglycerides, mmol/l (mean ± SD)	1.6 ± 4.1	1.6 ± 1.1	0.901
Total cholesterol, mmol/l (mean ± SD)	3.9 ± 0.9	3.9 ± 1.1	0.365
HDL-cholesterol, mmol/l (mean ± SD)	1.1 ± 0.3	1.0 ± 0.3	0.019
LDL-cholesterol, mmol/l (mean ± SD)	2.3 ± 0.8	2.33 ± 0.9	0.211
Creatinine, *μ*mol/l (mean ± SD)	96.6 ± 68.1	138.0 ± 106.8	<0.001
PT, *s* (mean ± SD)	13.3 ± 2.5	14.1 ± 4.7	0.117
APTT, *s* (mean ± SD)	42.3 ± 21.9	36.8 ± 11.9	0.101
CK, U/l (mean ± SD)	350.8 ± 714.9	294.6 ± 524.1	0.561
CK-Mb, U/l (mean ± SD)	42.0 ± 102.8	38.4 ± 104.7	0.681
TnT, pg/ml (median, IQR)	20.1 (11.3–1268.5)	46.7 (12.2–1418.3)	0.365
hs-CRP, mg/l (median, IQR)	4.2 (1.2–23.2)	7.0 (1.8–20.5)	0.173
NT-proBNP, pg/ml (median, IQR)	887.3 (290.0–2656.2)	1704.0 (536.6–5077.2)	<0.001
EF, % (mean ± SD)	54 ± 10.1	52 ± 11.8	0.072

sUA: serum uric acid; WBC: white blood cells; HbA1c: glycated hemoglobin; ALT: alanine aminotransferase; AST: aspartate aminotransferase; HDL: high-density lipoprotein; LDL: low-density lipoprotein; PT: prothrombin time; APTT: activated prothrombin time; CK: creatine kinase; CK-Mb: creatine kinase Mb chain; TnT: troponin *T*; hs-CRP: high-sensitivity C-reactive protein; NR-proBNP: N-terminal prohormone of brain natriuretic peptide; EF: ejection fraction.

**Table 3:** In-hospital events and follow-up for patients with normal sUA or hyperuricemia.

	sUA normal (*n* = 460)	Hyperuricemia (*n* = 251)	*P*
*In-hospital events*			
Heart failure, *n* (%)	68 (15.0)	51 (20.8)	0.053
Bleeding, *n* (%)	34 (7.5)	27 (11.1)	0.113
VT, *n* (%)	31 (6.8)	28 (11.3)	0.040
Stroke, *n* (%)	6 (1.3)	1 (0.4)	0.244
Death, *n* (%)	7 (3.7)	5 (4.8)	0.642

*One-year follow-up*			
Revascularization	6 (1.5)	5 (2.3)	0.432
Readmission, *n* (%)	158 (36.7)	97 (42.7)	0.129
Stroke, *n* (%)	24 (5.9)	7 (3.3)	0.159
Bleeding, *n* (%)	294 (68.2)	154 (67.2)	0.801
Death, *n* (%)	77 (16.7)	58 (23.1)	0.039

sUA: serum uric acid; VT: ventricular tachycardia.

**Table 4:** Differences between women and men.

Parameters	Women				Men				^*∗*^ *P*
	All (*n* = 273)	sUA normal (*n* = 164)	Hyperuricemia (*n* = 109)	^#^ *P*	All (*n* = 438)	sUA normal (*n* = 296)	Hyperuricemia (*n* = 142)	^&^ *P*	
*Demographics and medical history*									
Age, years (median, IQR)	78 (76–81)	78 (76–80)	78 (76–81)	0.510	78 (76–80)	78 (76–80)	78 (76–80)	0.455	0.381
Body mass index (median, IQR)	22.7 (20.6–25.4)	22.8 (20.6–25.1)	22.5 (20.5–26.3	0.644	23.1 (20.9–25.4)	22.8 (20.9–25.5)	23.7 (20.8–26.3)	0.065	0.427
Diabetes mellitus, *n* (%)	96 (35.2)	57 (34.8)	39 (35.8)	0.862	116 (26.5)	81 (27.4)	35 (24.6)	0.546	0.014
Hypertension, *n* (%)	217 (79.5)	126 (76.8)	91 (83.5)	0.182	319 (72.8)	208 (70.3)	111 (78.2)	0.02	0.056
Atrial fibrillation, *n* (%)	30 (11.2)	16 (9.9)	14 (13.0)	0.440	53 (12.3)	27 (9.2)	26 (18.7)	0.005	0.656
CKD ≥3	13 (4.8)	3 (1.8)	10 (9.2)	0.005	26 (5.9)	14 (4.7)	12 (8.5)	0.125	0.506
Stroke, *n* (%)	32 (11.9)	19 (11.8)	13 (12.1)	0.931	71 (16.5)	51 (17.5)	20 (14.5)	0.438	0.098
Previous chest pain, *n* (%)	181 (67.5)	111 (68.9)	70 (65.4)	0.546	286 (66.2)	188 (64.2)	98 (70.5)	0.193	0.716
Previous PCI, *n* (%)	36 (13.5)	24 (15.0)	12 (11.2)	0.375	78 (18.1)	58 (19.8)	20 (14.4)	0.172	0.112
Previous CABG, *n* (%)	3 (1.1)	2 (1.2)	1 (0.9)	0.815	10 (2.3)	7 (2.4)	3 (2.2)	0.882	0.255
Current smoking, *n* (%)	14 (5.4)	7 (4.4)	7 (6.8)	0.407	239 (56.5)	164 (57.3)	75 (54.7)	0.614	<0.001

*Clinical presentation*									
Hyperuricemia, *n* (%)	109 (39.9)				142 (32.4)				0.042
Severe heart failure, *n* (%)	155 (56.8)	83 (50.6)	72 (66.1)	0.012	201 (46.0)	120 (40.7)	81 (57.0)	0.001	0.005
STEMI, *n* (%)	55 (20.5)	38 (23.6)	17 (15.9)	0.126	119 (27.9)	77 (26.9)	42 (30.0)	0.506	0.028
NSTE-ACS, *n* (%)	213 (79.5)	123 (76.4)	90 (84.1)	0.126	307 (72.1)	209 (73.1)	98 (70.0)	0.506	0.028

*Laboratory data*									
WBC, 10^9^/L (mean ± SD)	7.4 ± 3.3	7.3 ± 3.4	7.5 ± 3.1	0.655	7.2 ± 3.2	7.1 ± 3.2	7.5 ± 3.2	0.287	0.568
Hemoglobin, g/L (mean ± SD)	110.4 ± 18.3	112.4 ± 16.5	107.3 ± 20.5	0.025	121.8 ± 20.2	123.1 ± 20.0	118.9 ± 21.1	0.039	<0.001
Platelets, 10^9^/L (mean ± SD)	202.9 ± 64.9	208.5 ± 64.6	194.7 ± 64.8	0.090	174.3 ± 61.4	117.9 ± 61.4	166.7 ± 61.0	0.080	<0.001
Fasting glucose, mmol/l (median, IQR)	6.0 (4.9–7.6)	6.1 (5.0–7.9)	5.9 (4.9–6.9)	0.255	5.7 (4.8–7.1)	5.7 (4.8–7.1)	5.6 (4.8–6.7)	0.467	0.181
HbA1C, % (median, IQR)	6.7 (6.0–7.4)	6.5 (5.9–7.5)	6.7 (6.0–7.0)	0.891	6.1 (5.6–6.5)	6.2 (5.7–6.8)	5.8 (5.5–6.2)	0.078	0.005
ALT, U/l (median, IQR)	17.5 (11.7–28.3)	17.6 (11.8–28.2)	17.5 (11.5–29.6)	0.932	19.9 (13.6–33.5)	20.2 (13.9–33.2)	19.6 (13.3–34.8)	0.738	0.012
AST, U/l (median, IQR)	22.6 (17.1–39.5)	22.0 (16.3–47.0)	22.8 (19.2–36.8)	0.728	22.6 (17.8–49.9)	23.5 (17.8–55.5)	21.5 (17.4–38.0)	0.742	0.579
Albumin, g/L (mean ± SD)	35.1 ± 4.5	35.1 ± 4.2	35.1 ± 4.8	0.942	35.1 ± 4.1	35.2 ± 3.9	34.9 ± 4.4	0.516	0.902
Triglycerides, mmol/l (mean ± SD)	1.6 ± 1.0	1.5 ± 0.9	1.7 ± 1.2	0.323	1.6 ± 4.2	1.6 ± 5.1	1.5 ± 1.0	0.740	0.960
Total cholesterol, mmol/l (mean ± SD)	4.1 ± 1.0	4.1 ± 1.0	4.1 ± 1.1	0.913	3.8 ± 0.9	3.7 ± 0.9	3.8 ± 1.0	0.400	<0.001
HDL-cholesterol, mmol/l (mean ± SD)	1.1 ± 0.3	1.1 ± 0.3	1.1 ± 0.3	0.288	1.0 ± 0.3	1.1 ± 0.3	1.0 ± 0.2	0.012	0.002
LDL-cholesterol, mmol/l (mean ± SD)	2.4 ± 0.9	2.4 ± 0.9	2.5 ± 1.0	0.575	2.2 ± 0.8	2.2 ± 0.8	2.3 ± 0.9	0.373	0.002
Creatinine, *μ*mol/l (mean ± SD)	89.9 ± 54.1	76.0 ± 35.3	111.1 ± 69.2	<0.001	124.4 ± 98.6	108.0 ± 78.5	158.3 ± 124.4	<0.001	<0.001
sUA, *μ*mol/l (mean ± SD)	349.2 ± 119.1	274.4 ± 52.0	462.0 ± 101.3	<0.001	381.4 ± 110.1	322.6 ± 61.9	504.1 ± 84.3	<0.001	<0.001
PT, *s* (mean ± SD)	13.7 ± 4.3	13.1 ± 1.5	147 ± 6.5	0.100	13.5 ± 2.8	13.4 ± 2.9	13.6 ± 2.4	0.715	0.674
APTT, *s* (mean ± SD)	39.5 ± 14.4	40.1 ± 15.5	38.5 ± 12.8	0.667	41.4 ± 22.2	43.5 ± 24.7	35.4 ± 11.1	0.109	0.562
CK-Mb, U/l (mean ± SD)	35.0 ± 71.4	37.6 ± 81.5	30.9 ± 52.3	0.475	44.3 ± 119.0	44.4 ± 113.0	44.1 ± 131.1	0.982	0.266
TnT, pg/ml (median, IQR)	19.3 (9.7–603.3)	16.9 (9.5–847.0)	27.8 (9.0–603.3)	0.819	33.2 (12.3–1461.0)	21.4 (11.6–1305.5)	56.0 (13.6–3185.8)	0.290	0.141
hs-CRP, mg/l (median, IQR)	4.3 (1.3–23.2)	4.3 (1.2–25.5)	5.3 (1.6–20.7)	0.967	5.1 (1.4–20.2)	4.2 (1.2–20.8)	7.5 (2.2–20.1)	0.076	0.849
NT-proBNP, pg/ml (median, IQR)	935.9 (290.4–3082.5)	718.0 (255.5–1999.1)	1619.2 (433.1–5058.7)	0.001	1337.0 (394.9–3737.7)	1037.0 (321.5–2917.2)	2055.0 (618.1–5153.8)	0.001	0.067
EF, % (mean ± SD)	53 ± 10.4	55 ± 9.2	51.6 ± 11.7	0.009	53 ± 10.9	53 ± 1.05	53 ± 11.9	0.754	0.460

*In-hospital management*									
Aspirin, *n* (%)	227 (83.2)	141 (86.0)	86 (78.9)	0.126	376 (86.2)	265 (89.9)	111 (78.2)	0.001	0.262
Clopidogrel, *n* (%)	234 (86.0)	141 (86.0)	93 (86.1)	0.975	391 (89.5)	266 (90.1)	125 (88.7)	0.699	0.168
ACEI/ARB, *n* (%)	168 (61.5)	105 (64.0)	63 (57.8)	0.300	268 (61.2)	189 (63.9)	79 (55.6)	0.099	0.925
Beta blocker, *n* (%)	206 (75.5)	123 (75.0)	83 (76.1)	0.829	306 (69.9)	207 (69.9)	99 (69.7)	0.964	0.106
Statin, *n* (%)	265 (97.8)	157 (96.9)	108 (99.1)	0.234	422 (96.3)	288 (97.3)	134 (94.4)	0.126	0.283
Diuretic, *n* (%)	128 (47.6)	67 (41.6)	61 (56.5)	0.017	210 (48.6)	133 (45.4)	77 (55.4)	0.052	0.791
PPI, *n* (%)	227 (84.4)	133 (82.6)	94 (87.0)	0.327	340 (78.7)	230 (78.5)	110 (79.1)	0.880	0.063
IABP, *n* (%)	7 (2.6)	3 (1.9)	4 (3.7)	0.353	18 (4.2)	12 (4.1)	6 (4.4)	0.890	0.255

*In-hospital events*									
Heart failure, *n* (%)	42 (15.7)	22 (13.7)	20 (18.9)	0.253	77 (17.9)	46 (15.8)	31 (22.3)	0.100	0.458
Bleeding, *n* (%)	22 (8.2)	11 (6.8)	11 (10.4)	0.303	39 (9.1)	23 (7.9)	16 (11.6)	0.210	0.706
VT, *n* (%)	23 (8.6)	10 (6.2)	13 (12.0)	0.094	36 (8.3)	21 (7.2)	15 (10.8)	0.203	0.920
Stroke, *n* (%)	5 (1.9)	4 (2.5)	1 (0.9)	0.354	2 (0.5)	2 (0.7)	0 (0.0)	0.329	0.071
Death, *n* (%)	4 (3.5)	1 (1.4)	3 (7.0)	0.119	8 (4.4)	6 (5.0)	2 (3.3)	0.587	0.693

*One-year follow-up*									
Revascularization	4 (1.6)	1 (0.6)	3 (3.2)	0.120	7 (1.9)	5 (2.0)	2 (1.7)	0.841	0.801
Readmission, *n* (%)	96 (36.9)	57 (35.8)	39 (38.6)	0.653	159 (39.9)	101 (37.1)	58 (46.0)	0.092	0.436
Stroke, *n* (%)	13 (5.2)	7 (4.5)	6 (6.4)	0.513	18 (4.8)	17 (6.7)	1 (0.8)	0.013	0.827
Bleeding, *n* (%)	180 (69.2)	109 (69.0)	71 (69.6)	0.916	268 (67.0)	185 (67.8)	83 (65.4)	0.633	0.549
Death, *n* (%)	40 (14.7)	16 (9.8)	24 (22.0)	0.005	95 (21.7)	61 (20.6)	34 (32.4)	0.428	0.020

sUA: serum uric acid; IQR: interquartile range; CKD: chronic kidney disease; PCI: percutaneous coronary intervention; CABG: coronary artery bypass graft; SBP: systolic blood pressure; DBP: diastolic blood pressure; STEMI: ST-elevated myocardial infarction; NSTE-ACS: non-ST-elevation acute coronary syndrome; WBC: white blood cells; HbA1c: glycated hemoglobin; ALT: alanine aminotransferase; AST: aspartate aminotransferase; HDL: high-density lipoprotein; LDL: low-density lipoprotein; PT: prothrombin time; APTT: activated prothrombin time; CK: creatine kinase; CK-Mb: creatine kinase Mb chain; TnT: troponin *T*; hs-CRP: high-sensitivity C-reactive protein; NR-proBNP: N-terminal prohormone of brain natriuretic peptide; EF: ejection fraction; ACEI: angiotensin-converting enzyme inhibitor; ARB: angiotensin receptor blocker; PPI: proton pump inhibitor; IABP: intra-aortic balloon pump; VT: ventricular tachycardia.

## Data Availability

The data used to support the findings of this study are included within the supplementary information file.

## Additional Points

Elderly ACS patients with hyperuricemia have higher in-hospital events and 1-year all-cause mortality compared with those without hyperuricemia. Hyperuricemia is an independent risk factor for one-year all-cause mortality after discharge in elderly female patients with ACS, but not in elderly male patients with ACS.

## Supplementary Materials

Supplementary Materials The supplementary materials contain two tables. In-hospital management information is shown in Supplemental Table 1, and the independent determinants of 1-year all-cause mortality are shown in Supplemental Table 2. And the supplementary Excel file is the datasheet used for the statistical analysis.
