# Supplementary material for: Hyperuricemia is a Risk Factor for One-Year Overall Survival in Elderly Female Patients with Acute Coronary Syndrome
Source: Cardiovasc Ther. 2020 Feb 22;2020:2615147. doi: 10.1155/2020/2615147 (PMC7057023; doi:10.1155/2020/2615147)
Supplement: Supplementary Materials — The supplementary materials contain two tables. In-hospital management information is shown in Supplemental Table 1, and the independent determinants of 1-year all-cause mortality are shown in Supplemental Table 2. And the supplementary Excel file is the datasheet used for the statistical analysis. [file 2615147.f1.zip › 2615147.f1/Supplemental Tables.pdf]

Supplemental Table 1. In-hospital management for patients with normal sUA or Hyperuricemia

	sUA normal (n=460)	Hyperuricemia (n=251)	*P*
**Medical therapy**			
Aspirin, n (%)	406 (88.6)	197 (78.5)	<0.001
Clopidogrel, n (%)	407 (88.5)	218 (87.6)	0.715
ACEI/ARB, n (%)	294 (63.9)	142 (56.6)	0.055
Beta blocker, n (%)	330 (71.7)	182 (72.)	0.827
Statin, n (%)	445 (97.2)	242 (96.4)	0.583
Diuretic, n (%)	200 (44.1)	138 (55.9)	0.003
PPI, n (%)	363 (80.0)	204 (82.6)	0.397
**IABP, n (%)**	15 (3.3)	10 (4.1)	0.598
**Hemodialysis, n (%)**	2 (1.4)	2 (2.6)	0.532

sUA: serum uric acid; ACEI: angiotensin-converting enzyme inhibitor; ARB: angiotensin receptor blocker; PPI: proton pump inhibitor; IABP: intra-aortic balloon pump

Supplemental Table 2. Multivariable analyses of the factors associated with 1-year mortality

	Unadjusted			Model 1			Model 2			Model 3		
	OR	95% CI	*P*	OR	95% CI	*P*	OR	95% CI	*P*	OR	95% CI	*P*
Women												
sUA ≥357 µmol/l vs. <357 µmol/l	2.612	1.315-5.189	0.006	2.438	1.215-4.894	0.012	2.643	1.092-6.400	0.031	2.539	1.001-6.453	0.050
1-SD increase in sUA	1.005	1.002-1.007	0.001	1.004	1.002-1.007	0.001	1.005	1.001-1.008	0.012	1.004	1.000-1.008	0.043
Men												
sUA ≥416 µmol/l vs. <416 µmol/l	1.264	0.775-2.064	0.348	1.186	0.733-1.918	0.487	0.865	0.438-1.708	0.675	0.931	0.466-1.858	0.839
1-SD increase in sUA	1.001	1.000-1.004	0.096	1.002	0.999-1.004	0.144	1.002	0.999-1.005	0.286	1.002	0.999-1.005	0.277
Total												

sUA ≥416/357 µmol/l vs. <416/357 µmol/l	1.512	1.028-2.225	0.036	1.450	0.981-2.142	0.062	1.349	0.799-2.277	0.262	1.326	0.773-2.273	0.305
1-SD increase in sUA	1.003	1.001-1.004	0.001	1.003	1.001-1.005	0.007	1.003	1.001-1.005	0.004	1.003	1.001-1.005	0.014

Sample size, n=711. Data are expressed as OR±95% CIs (reported in parentheses) as assessed by univariate (unadjusted) or multivariate logistic regression analyses.

OR: odds ratio; CI: confidence interval; sUA: serum uric acid; SD: standard deviation.

In these logistic regression models, sUA level was included as either a continuous variable (for each 1-SD increase) or a categorical variable (stratifying by presence of hyperuricemia). Hyperuricemia was defined as a sUA level≥ 416 µmol/l in men and ≥357 µmol/l in women.

Other covariates included in multivariate logistic regression models were model 1: age; model 2: age, BMI, current smoking, hypertension, and diabetes mellitus; model 3: adjustment for variables included age, BMI, current smoking, PCI or CABG history, hypertension, diabetes mellitus, CKD≥3, and stroke.
